# Versatile Mitogenic and Differentiation‐Inducible Layer Formation by Underwater Adhesive Polypeptides

**DOI:** 10.1002/advs.202100961

**Published:** 2021-06-26

**Authors:** Seiichi Tada, Xueli Ren, Hongli Mao, Yun Heo, Shin‐Hye Park, Takashi Isoshima, Liping Zhu, Xiaoyue Zhou, Reiko Ito, Shino Kurata, Megumi Osaki, Eiry Kobatake, Yoshihiro Ito

**Affiliations:** ^1^ Emergent Bioengineering Materials Research Team RIKEN Center for Emergent Matter Science 2‐1 Hirosawa Wako Saitama 351‐0198 Japan; ^2^ Nano Medical Engineering Laboratory RIKEN Cluster for Pioneering Research 2‐1 Hirosawa Wako Saitama 351‐0198 Japan; ^3^ Department of Environmental Chemistry and Engineering Interdisciplinary Graduate School of Science and Engineering Tokyo Institute of Technology Midori‐ku Yokohama 226–8502 Japan; ^4^ Support Unit for Bio‐Material Analysis Research Resources Division RIKEN Center for Brain Science 2‐1 Hirosawa Wako Saitama 351‐0198 Japan

**Keywords:** 3, 4‐dihydroxyphenylalanine, adhesive growth factor, epidermal growth factor, hydroxyapatite, insulin‐like growth factor, polystyrene, titanium

## Abstract

Artificial materials have no biological functions, but they are important for medical devices such as artificial organs and matrices for regenerative medicine. In this study, mitogenic and differentiation‐inducible materials are devised via the simple coating of polypeptides, which contain the sequence of epidermal growth factor or insulin‐like growth factor with a key amino acid (3,4‐dihydroxyphenylalanine) of underwater adhesive proteins. The adhesive polypeptides prepared via solid‐phase synthesis form layers on various substrates involving organic and inorganic materials to provide biological surfaces. Through the direct activation of cognate receptors on interactive surfaces, the materials enable increased cell growth and differentiation compared to that achieved by soluble growth factors. This superior growth and differentiation are attributed to the long‐lasting signal transduction (triggered by the bound growth factors), which do not cause receptor internalization and subsequent downregulation.

## Graphical abstract

3,4‐dihydroxyphenylalanine‐containing peptides form bioactive layers on organic and inorganic material surfaces. On the adhesive growth factor nanolayers, cells receive long‐term biological signals from immobilized growth factors, leading to improved cell growth and differentiation.

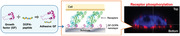

## 1 Introduction

Various biomaterials, including organic and inorganic matters, have been developed for artificial organs or tissue engineering. Non‐biofouling or cell‐adhesive surfaces have been devised for use in the human body. However, the addition of biological activity to biomaterials—such as growth enhancement—is difficult. Recent studies have revealed that the immobilization of growth factor protein enables the stimulation of the mitogenic and differentiating signals from biomaterials.^[^ ^1^, ^2^, ^3^, ^4^, ^5^ ^]^ Protein tethering has been realized using various methods; however, the immobilization methods for biomacromolecules on inorganic surfaces with maintained bioactivity and molecular orientation are still limited.^[^ ^5^, ^6^, ^7^, ^8^, ^9^, ^10^, ^11^ ^]^ Therefore, we aimed to develop an adhesive polypeptide that would form a nanolayer on surfaces and provide mitogenic and differentiating activities.

**Figure 1:**
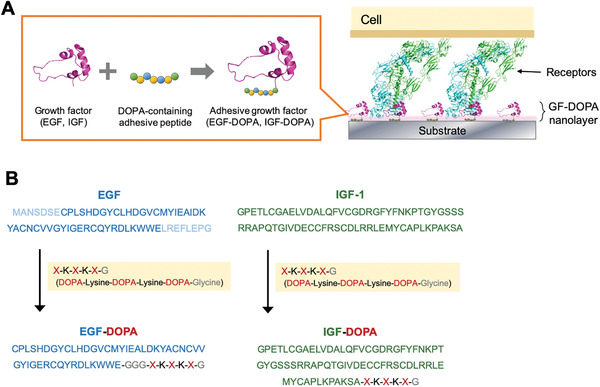
Design of adhesive growth factors (EGF‐DOPA and IGF‐DOPA). A) Illustration of layer‐forming growth factors on the substrate to generate bioactive surfaces. B) Sequences of EGF‐DOPA and IGF‐DOPA.

3,4‐Dihydroxyphenylalanine (DOPA) is a noncanonical amino acid and is found in mussel foot proteins (Mfps). DOPA plays an important role in the binding of marine mussels to solid surfaces by providing a wide range of interactions, including hydrogen and coordination bonding via its catechol group and cation–*π* interaction with the catechol group and the amine group in the lysine in Mfps.^[^ ^12^, ^13^, ^14^, ^15^, ^16^ ^]^ Therefore, DOPA has been applied to the immobilization of a wide range of functional molecules, such as bioactive macromolecules or antifouling compounds, on various material surfaces, including metallic (titanium (Ti), copper, and gold), inorganic (glass and hydroxyapatite (HA)) and organic (polystyrene (PS), poly(tetrafluoroethylene) and polyethylene terephthalate) materials.^[^ ^12^, ^13^, ^14^, ^15^, ^16^, ^17^, ^18^, ^19^ ^]^ For immobilizing bioactive proteins, tyrosine residues in the protein can be converted to DOPA via oxidant or tyrosinase treatment. However, tyrosine oxidation process changes any positions of tyrosine in the protein into DOPA and results in poor orientation on bound surfaces and loss of biological activity in some cases. Thus, the DOPA molecule should be introduced into proteins in a site‐specific manner without affecting other amino acids. Here, we incorporated DOPA‐containing peptide moiety into epidermal growth factor (EGF) and insulin‐like growth factor (IGF) via the solid‐phase peptide synthesis (SPPS) method for preparing biologically active layers capable of inducing cell mitosis or differentiation on multiple types of biomaterials: Ti, HA, and PS (**Figure** **1**A,B). The DOPA adhesion mechanism was applied for the enhancement effect of cell growth and differentiation, which are related to the cellular gene expression level through signal transduction inside the cell, on various materials.

## 2 Results

### 2.1 Growth Factor Layers were Formed on Organic and Inorganic Material Surfaces Via a DOPA‐Lysine Peptide

For an adhesive peptide sequence with DOPA moiety, we selected the sequence XKXKXG, where *X*, *G*, and *K* represent DOPA, glycine, and lysine, respectively. This sequence was originally designed by Messersmith et al.—inspired by Mfps secreted by mussels.^[^ ^17^ ^]^ Mfps tightly adheres to inorganic surfaces via DOPA and other amino acids (such as lysine, serine, and cysteine, with various interactions) through hydrogen bonding; coordination bonding; and electrostatic, hydrophobic, and cation–*π* interactions.^[^ ^12^, ^13^, ^14^, ^15^, ^16^ ^]^ The contribution of each interaction depends on the surrounding conditions, including pH and ionic strength. Under the moderate pH condition without causing severe loss of protein activity, for example, pH 4–9, hydrogen and coordination bonding of DOPA, cation–*π* interaction between lysine residues and aromatic groups, and polymerization of catechol moiety and Michael addition or Schiff base reaction with amino groups contribute to surface binding and thin‐layer formation of DOPA‐containing peptides and proteins.^[^ ^14^, ^15^, ^17^, ^18^, ^19^ ^]^ In this study, the XKXKXG sequence was compared to the other three sequences, XSXSXG, XGXGXG, and YKYKYG, whose *S* and *Y* represent serine and tyrosine, for their respective potentials for hydrogen bond formation. The amount of the peptide bound to various materials surfaces (Figure S1A, Supporting Information), the effect of pH on the amount of peptide bound to the surfaces (Figure S1B, Supporting Information), and the potential reactivity of the bound peptide under the long‐term incubation (Figure S1C–E, Supporting Information) were estimated. XKXKXG demonstrated the best binding property and reactivity among those peptides as confirmed in our previous work.^[^ ^18^ ^]^ Because the bound amount of XKXKXG increased in basic pH conditions, the sequence binding was attributed to multiple types of interaction other than hydrogen bonding (Figure S1B, Supporting Information), such as cation–*π* interaction and catechol polymerization. We selected this sequence for synthesizing adhesive growth factors.

We introduced this DOPA‐lysine peptide sequence to growth factors via solid‐phase synthesis of whole protein sequences. Because the length of peptides synthesized using this method is usually limited, it is preferable to design as short a peptide‐linked protein as possible. Here, we employed the biologically active domain of EGF comprising 46 amino acid residues, which can be maintained via truncation, as previously reported,^[^ ^19^, ^20^, ^21^ ^]^ to construct the adhesive growth factor (Figure 1B). We connected the binding sequence, XKXKXG, with the active domain of EGF through a glycine–glycine–glycine spacer to construct an EGF derivative (EGF‐DOPA) with a universal binding affinity to various substrates. For terminal modification of EGF, both N‐ and C‐terminal modifications—despite the possibility of activity loss induced by the conformational changes—have been reported. The crystal structure analysis of the complex of EGF and EGF receptors suggested that the N‐terminus of EGF does not interact with the EGF receptor,^[^ ^22^ ^]^ however, the modified EGF maintains a certain level of biological activity whichever terminus was modified via various binding domains.^[^ ^10^, ^11^, ^12^, ^13^, ^14^, ^15^, ^16^, ^17^, ^18^, ^19^ ^]^ In this study, we, therefore, combined the binding peptide on the C‐terminal of EGF. For IGF, we used the full length of the growth factor sequence for synthesizing an adhesive growth factor to avoid the loss of biological activity.^[^ ^23^ ^]^ Cryoelectron microscopy analysis suggests that the immobilization of IGF via C‐terminal modification with a short peptide does not hinder the interaction of IGF and dimer of IGF receptors, as shown in Figure 1A.^[^ ^24^ ^]^ Therefore we conjugated the same binding sequence—XKXKXG—on the C‐terminus of IGF (Figure 1B). We synthesized the designed polypeptides via the solid‐phase method, as described in the experimental section and supporting information (Figure S2, Supporting Information).

**Figure 2:**
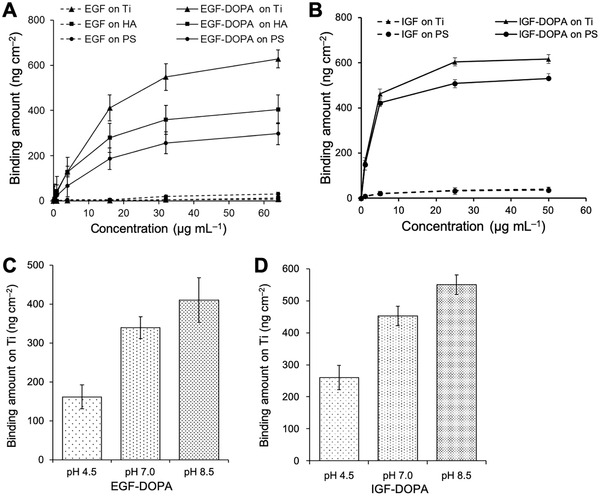
Binding analysis of adhesive growth factors (EGF‐DOPA and IGF‐DOPA) to material surfaces. A) Binding amounts of EGF and EGF‐DOPA on titanium (Ti), hydroxyapatite (HA), and polystyrene (PS) surfaces at different concentrations under pH 8.5 via QCM‐D analysis. B) IGF‐DOPA binding analysis with QCM‐D on Ti and PS, in a similar manner to that used with EGF‐DOPA. C,D) Binding amounts of EGF‐DOPA and IGF‐DOPA, respectively, on Ti surfaces under different pH values. The concentration of the solutions was 16 µg·mL^−1^. Data are shown as means ± s. d. (n = 3).

We confirmed the binding affinities of DOPA‐conjugated growth factors and wild‐type growth factors on representative inorganic and organic materials, including Ti, HA, and PS, using a quartz crystal microbalance with dissipation monitoring (QCM‐D) (**Figure** **2**A,B). EGF‐DOPA showed a strong binding affinity with these three substrates, whereas unconjugated EGF showed weak or no binding affinity to them (Figure 2A; Figure S3A,B, Supporting Information). IGF‐DOPA also bound to Ti and PS surfaces robustly, whereas the unconjugated IGF did not bind to the surfaces (Figure 2B; Figure S3B, Supporting Information). The amount of DOPA‐growth factor binding to the material surface varies; the highest amount binds to the Ti surface, followed by the HA and PS surfaces. Such difference is attributed to the initial interaction between the catechol groups and material surfaces. A certain number of catechol groups can interact with the Ti surface via hydrogen and coordination bonding even at pH 8.5 because the pKa_1_ of 3’‐hydroxyl group of the catechol moiety is approximately 8.7.^[^ ^17^, ^18^ ^]^ On the other hand, the catechol groups can form hydrogen bonding but not coordination bonding with the HA surface. Meanwhile, a certain number of hydroxyl and carbonyl groups are formed on the tissue culture‐treated PS surface by plasma treatment, thus enabling hydrogen bonding with the catechol groups and the primary amine groups of the lysine residues. Also, the density of hydroxyl and carbonyl groups on the PS surface would be lower than the density of Ca^2+^ on the HA surface. Thus, the amount of EGF‐DOPA binding will be highest on the Ti surfaces, followed by the HA and PS surfaces.

Furthermore, the bound EGF‐DOPA and IGF‐DOPA on the material surface hardly dissociated from the surfaces even after washing with PBS (Figure S3A–C, Supporting Information). Similar to the XKXKXG peptide sequence, DOPA‐conjugated growth factors exhibited binding affinity to the substrate in a pH‐dependent manner (Figure 2C,D). The strongest binding occurred under alkaline conditions (pH 8.5). We attributed the enhanced binding affinity of DOPA‐growth factors mainly to the reaction between the catechol moieties of DOPA and the amine groups of the lysine residues because this reaction is accelerated in high pH, in contrast to the formation of hydrogen or coordination bonding between the catechol groups and the material surfaces.^[^ ^14^, ^15^ ^]^ Even at pH 4.5, EGF‐DOPA was immobilized with a density of 162 ng cm^−2^ (1.5 × 10^5^ molecules µm^−2^) on Ti surface, which is approximately 24000 times higher than the density of EGF receptor (EGFR; 6.3 molecules µm^−2^) on HeLa cell surface.^[^ ^25^ ^]^ EGFR stimulation experiments using EGF‐immobilized gold nanoparticles suggested that nanoparticles with EGF at a density of 1.2 × 10^4^ molecules µm^−2^ strongly induced EGFR phosphorylation in HeLa cells.^[^ ^26^ ^]^ These results indicate that the DOPA‐lysine peptide sequence immobilized a sufficient amount of growth factors to stimulate the corresponding receptors. We further confirmed the binding of EGF‐DOPA on Ti surfaces via immunofluorescent staining of growth factors using an anti‐EGF antibody (Figure S3D, Supporting Information). We detected signals only from the Ti‐surface bound with EGF‐DOPA. This suggested that the EGF layer was formed on the surface by the DOPA moiety and that the EGF moiety was not fully buried in the layer, rather being displayed on the surface such that the antibodies could access their targets.

We analyzed the physical properties of growth factor layers using atomic force microscopy (AFM) and ellipsometry (Figure S4, Supporting Information). First, we investigated the topography of the EGF‐DOPA‐modified surfaces via AFM (Figure S4A, Supporting Information). We observed uniformly distributed nanostructures on the surfaces bound by EGF‐DOPA under pH 4.5. On the other hand, there were larger nanostructures under pH 8.5, which might be attributed to EGF‐DOPA aggregation formed by the polymerization of catechol moieties. Because the height of these nanostructures was less than 4 nm (Figure S4A, Supporting Information), the influence of this surface structure itself would be negligible for the cells. Thus, the immobilized growth factors were expected to affect the cell behavior most.^[^ ^27^ ^]^

We investigated the thickness of EGF‐DOPA and IGF‐DOPA bound on the substrates through ellipsometry measurement (Figure S4B,C, Supporting Information). For EGF‐DOPA, the thickness increased with increasing EGF‐DOPA concentration and reached a plateau at high EGF‐DOPA concentrations under the condition of pH 4.5. However, the thickness of EGF‐DOPA under the condition of pH 8.5 continued to increase with increasing EGF‐DOPA concentration, suggesting a multilayer formation of EGF‐DOPA on the substrate. For IGF‐DOPA, the thickness almost reached a plateau in the range of 5–42 µg mL^−1^ at pH 4.5. At 84 µg·mL^−1^ of IGF‐DOPA, the thickness increased again. De Crescenzo et al. reported that the EGF layer on a self‐assembled monolayer in an aligned manner using coiled‐coil interaction led to a net increase in the layer thickness (0.8 ± 0.2 nm as dry thickness),^[^ ^28^ ^]^ which is consistent with our result of the thickness increase after EGF‐DOPA immobilization at pH 4.5. This consistency suggests that EGF‐DOPA did not accumulate significantly at pH 4.5 but accumulated at pH 8.5 in a multilayer manner. Moreover, IGF‐DOPA showed a similar thickness under a low‐concentration condition at pH 4.5 and a thickness increase at a high concentration, suggesting thin‐layer formation with the saturated binding of IGF‐DOPA and subsequent stacking at high concentrations of IGF‐DOPA.

### 2.2 Cell Proliferation and Differentiation Induced by Surface‐Binding Growth Factors

**Figure 3:**
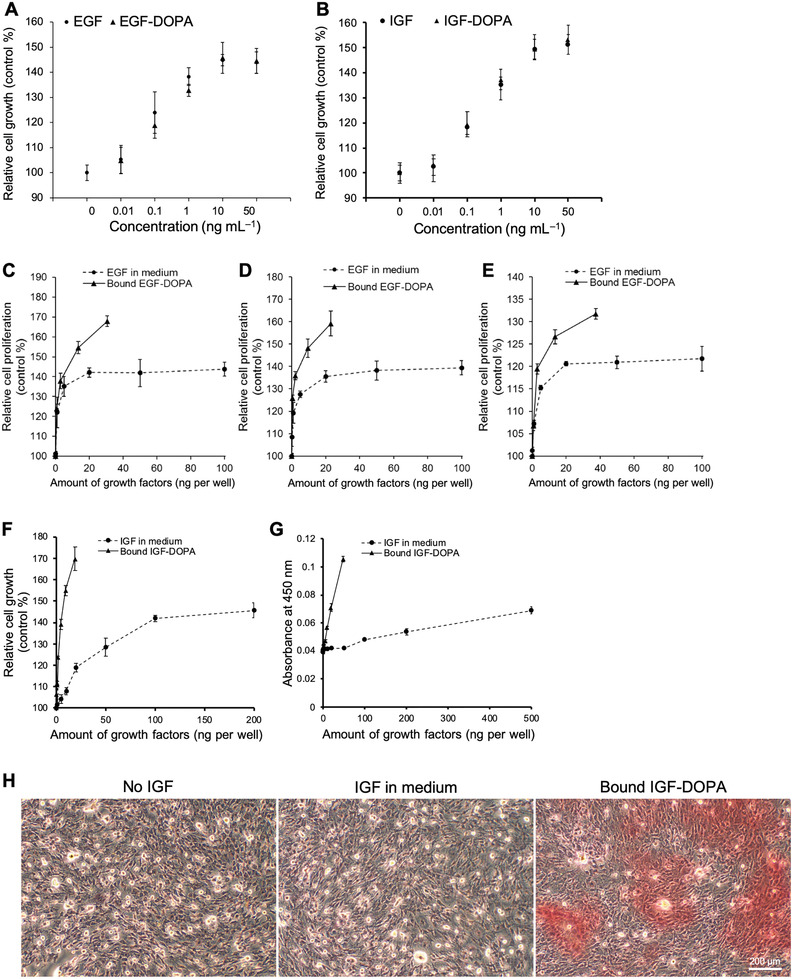
Biological activity of EGF‐DOPA and IGF‐DOPA. A,B) WST‐8 assay of cells cultured using EGF‐DOPA (A) and IGF‐DOPA (B) in the medium at different concentrations. C–E) WST‐8 assay of cells cultured using soluble EGF in medium and bound EGF‐DOPA on titanium (Ti, C), hydroxyapatite (HA, D), and polystyrene (PS, E) surfaces. F) WST‐8 assay of cells cultured using soluble IGF in medium and bound IGF‐DOPA on Ti surface. G,H) Differentiation of MC3T3‐E1 cells on IGF‐DOPA‐coated surface. (G) Quantification of Alizarin Red S staining of MC3T3‐E1 cells in the presence of soluble IGF or immobilized IGF‐DOPA. (H) Phase‐contrast images of the differentiated cells triggered by soluble IGF or bound IGF‐DOPA (50 and 47.5 ng/well, respectively). Data are shown as means ± s. d. (n = 3).

We elucidated the biological activities of DOPA‐conjugated growth factors via in vitro cellular analyses (**Figure** **3**). We investigated the mitogenic activities of EGF‐DOPA and IGF‐DOPA with NRK49F cells in a culture medium (Figure 3A,B) and detected no significant difference between the normal and DOPA‐conjugated growth factors, indicating that the modification with the binding DOPA sequence did not affect their biological activity.

We compared the growth of cells cultured using soluble EGF in medium and bound EGF‐DOPA on various material surfaces (Figure 3C–E). We used cells cultured on a bare Ti surface without EGF as a reference and considered them as displaying 100% proliferation in our calculations. Although we detected no significant difference between EGFs with and without DOPA in dissolved conditions (Figure 3A), the surface‐immobilized EGF‐DOPA was more active than the unconjugated EGF dissolved in a medium (Figure 3C; Figure S5, Supporting Information). Smaller amounts of bound EGF‐DOPA in culture showed greater effects on cell growth than those of soluble, normal EGF in the medium. We obtained similar results when culturing cells on HA and PS surfaces bound with EGF‐DOPA (Figure 3D,E). We previously investigated the influence of the surface coated with dopamine, a derivative of DOPA on the cell growth, and found that it was negligible.^[^ ^29^ ^]^ Therefore it is considered that the present enhancement effect was contributed from the part of the polypeptide.

For IGF‐DOPA, we investigated cell proliferation and differentiation activities (Figure 3F–H), thus identifying the growth of NIH3T3 cells on Ti treated with IGF‐DOPA bound to the surfaces to be more active than free IGF‐1 (Figure 3F). Moreover, we investigated the differentiation of MC3T3‐E1 cells on an IGF‐DOPA surface by Alizarin Red S staining of calcium deposition, one of the markers of osteoblast differentiation (Figure 3G,H). We detected almost no stained area in the negative control cells without IGF‐1 14 days after cell seeding. However, cultured cells on the IGF‐DOPA‐treated Ti surface showed more amount of staining than those in a normal IGF‐containing medium (Figure 3G,H). We quantified the Alizarin Red staining level by the absorbance measurement of Alizarin Red‐solubilized extract. The absorbance of the IGF‐DOPA‐immobilized condition showed a higher value than that of the IGF‐treated surface after 14 days (Figure 3G). This result indicated that cell differentiation was accelerated by the immobilization of IGF via an adhesive moiety.

These observations can be attributed to the high local concentration of growth factors and multivalency of immobilized growth factor layers. Compared to the effects of soluble EGF, the long‐lasting cell activation by immobilized EGF may arise through the inhibition of down‐regulating mechanisms.^[^ ^28^, ^30^ ^]^ Activation is enhanced via co‐immobilization of growth factors with adhesion factors.^[^ ^27^ ^]^ Immobilized EGF also induced quantitative effects (such as enhanced growth) and qualitative effects (such as differentiation), distinct from those observed in soluble EGF for certain cells.^[^ ^31^ ^]^

### 2.3 EGF‐DOPA Activated Cell Membrane Receptors in the Long Term from Adhered Surface

**Figure 4:**
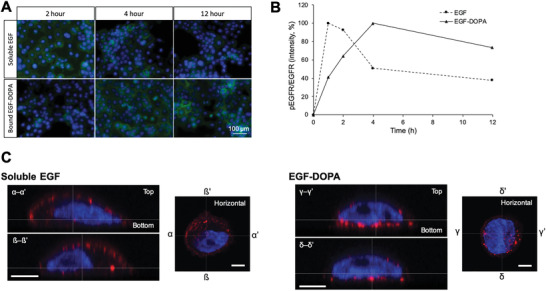
Activation of EGF receptors (EGFRs) by EGF‐DOPA. A) Immunofluorescence staining of the phosphorylated EGFRs triggered by soluble EGF and bound EGF‐DOPA. B) Quantitative analysis of the phosphorylated EGFRs triggered by soluble EGF and bound EGF‐DOPA. The ratio of phosphorylated EGFR intensity to total EGFR intensity was plotted from a series of single‐time course experiments of Western blotting. C) Distribution of phosphorylated EGFRs in A431 cells observed using a confocal microscope after 30‐min incubation. The images of *α*–*α*’, ß–ß’, *γ*–*γ*’, and *δ*–*δ*’ are vertical cross‐sectional views at corresponding positions shown as white‐dotted lines in horizontal views, respectively. The phosphorylated EGFRs were stained red by anti‐phosphorylated EGFR antibody and the nuclei were stained blue by DAPI. Scale bars: 5 µm.

To observe signal transduction from bound EGF‐DOPA, we investigated the intracellular distribution of the phosphorylated EGFR (**Figure** **4**). First, we measured the time course of activation of cellular signal transduction. In Figure 4A, we demonstrate the immunofluorescence staining for phosphorylation of the EGFRs triggered by EGF. The fluorescence signals from cells cultured using soluble EGF in the medium increased rapidly and decreased rapidly over time. However, the fluorescence signals from the cells cultured using bound EGF‐DOPA occurred gradually and lasted for a relatively longer duration. We quantified the phosphorylation level of EGFRs via Western blot analysis and detected <40% of phosphorylated EGFRs after the cells were cultured using soluble EGF for 12 h (Figure 4B). However, for bound EGF‐DOPA, we detected >70% of EGFRs in a phosphorylated state even after incubation for 12 h.

Moreover, we observed the intracellular distribution of phosphorylated EGFRs using a confocal laser scanning microscope to confirm that the immobilized EGF induced receptor phosphorylation (Figure 4C). Although phosphorylated EGFRs were spread throughout the cells cultured in a soluble EGF, certain cells cultured on EGF‐DOPA for 30 min showed a specific EGFR phosphorylation pattern, primarily on the basal side, attaching to the EGF‐DOPA surface. These results suggest that EGF molecules immobilized via DOPA interacted with cell surface EGFRs and that the phosphorylation signal was triggered by the growth factor‐immobilized substrate.

## 3 Discussion

In this study, EGF and IGF layers were formed on both organic and inorganic materials by conjugating the surface‐binding peptide sequence with a DOPA moiety. Many studies have used DOPA and dopamine molecules for immobilizing bioactive proteins, including growth factors and extracellular matrices (ECM).^[^ ^27^, ^28^, ^29^, ^30^, ^31^ ^]^ However, the orientation of immobilized protein cannot be controlled via catechol polymerization and Michael addition reaction because the primary amine groups can react in any position. Immobilization via DOPA formation through tyrosine oxidation leads to the poor orientation of proteins because of the uncontrollable transition of any tyrosine residues in immobilized proteins to DOPA. However, our method of synthesizing adhesive growth factors can clearly specify the DOPA position in the immobilized protein. Moreover, DOPA polymerization is suppressed under acidic conditions because of the slow oxidation of catechol moiety.^[^ ^14^ ^]^ Based on these properties of adhesive growth factors, we expect the formation of thin‐layer growth factors with molecular orientation to be possible. Our growth factor nanolayers exhibited sufficient biological activities to induce cell growth or differentiation (Figure 3), which suggests an effective display of growth factors on the nanolayer without severe activity loss.

Our growth factors with DOPA moiety formed bioactive layers displaying growth factors on various organic and inorganic surface materials—including Ti and HA (Figure 3; Figure S3, Supporting Information). Several studies have reported the immobilization of bioactive growth factors.^[^ ^27^, ^28^, ^29^, ^30^, ^31^, ^32^ ^]^ Unlike certain growth factor immobilization processes requiring surface precoating with linker molecules or ECM,^[^ ^27^, ^28^, ^30^ ^]^ our layer‐forming process can display growth factors via the simple immersion of material surfaces in a DOPA‐conjugated growth factor solution.

Several groups have investigated the mechanism behind the long‐lasting bioactivity of immobilized growth factors.^[^ ^33^, ^34^ ^]^ Arisaka et al. reported that internalization of basic FGF (bFGF) within cells on heparin‐functionalized surfaces is suppressed more clearly than that with soluble bFGF.^[^ ^34^ ^]^ They suggested that the stable binding of bFGF on heparinized surfaces inhibits the downregulation of FGF receptors, resulting in long‐term activation of intracellular signal transduction. In this study, we confirmed that compared with the effects of soluble EGF, the bound EGF‐DOPA causes a longer‐lasting activation of cellular signal transduction (Figure 4A,B). During the initial period of cell culture, immobilized EGF‐DOPA stimulated EGFRs expressed at the bottom of the cells (Figure 4C). Thus, in the long term, our EGF‐DOPA maintains biological activity even after immobilization and stimulated cells.

## 4 Conclusion

In summary, inspired by the underwater adhesive mussel protein, we developed EGF‐ and IGF‐derived layers. The synthesized growth factor derivatives showed a high binding affinity to a range of material surfaces and promoted cell growth more efficiently than soluble growth factors. IGF‐DOPA induced osteoblast differentiation to a greater degree than solubilized normal IGF. We conclude that such enhanced biological activities are caused by the prolonged activation of cell signal transduction. These modified growth factors provide new functional biomaterials suitable for surface modification and the development of medical devices.

## 5 Experimental Section

### Materials

Ti plates (Osaka Vacuum Industries, Osaka, Japan) were prepared via the vacuum deposition of Ti onto glass plates that were 15 mm in diameter and 1 mm in thickness using a 400‐nm (± 25%) electron beam after the glass plates were cleaned nine times via ultrasonication in ultrapure water and dried under a stream of heated air. The coated plate is referred to as Ti. HA plates with an 8‐mm width and 1‐mm thickness (Pentax, Tokyo, Japan) were procured. Tissue culture plates (BD Falcon, Corning, NY, USA) were used as hydrophilized PS surfaces.

### Peptide Synthesis

Four peptide sequences, DOPA (X)‐Lysine‐DOPA‐Lysine‐DOPA‐Glycine (XKXKXG), DOPA‐Glycine‐DOPA‐Glycine‐DOPA‐Glycine (XGXGXG), DOPA‐Serine‐DOPA‐Serine‐DOPA‐Glycine (XSXSXG), and Tyrosine‐Lysine‐Tyrosine‐Lysine‐Tyrosine‐Glycine (YKYKYG) with or without DOPA, were synthesized via SPPS to select the candidate with the best binding affinity. Peptide turbidity was measured using 100 µL of the sample at 2 mg mL^−1^ on a spectrophotometer (JASCO V‐550, Tokyo, Japan) at 450 nm.

The adhesive growth factor was prepared by adding the selected binding sequence to the truncated EGF's C‐terminus via SPPS using 9‐fluorenylmethoxycarbonyl (Fmoc) protected amino acids. The Fmoc‐Gly‐NovaSyn TGT (0.19 mmol g^−1^) resin (Novabiochem, San Diego, CA, USA), which was loaded with low‐density amino acids, was purchased. In addition to conventional Fmoc‐protected canonical amino acids, Fmoc‐DOPA (acetonide)‐OH (Merck, Darmstadt, Germany) was used.

On the contrary, the peptide synthesizer, ABI433A (Thermo Fisher Scientific, Waltham, MA, USA), was used to synthesize the EGF‐conjugated surface‐binding sequence. The polypeptide comprising 55 amino acids was synthesized according to the previous study.^[^ ^19^ ^]^ In short, a low‐density resin was used and considered the reaction conditions, including the agent amounts (the normal and double coupling was 1 and 2 mmol, respectively) and reaction times (normal and extended times were 10 and 30 min, respectively) (Figure S2A, Supporting Information). After purification using high‐performance liquid chromatography (HPLC) equipped with a YMC‐Pack PROTEIN‐RP column (250 × 4.6‐mm I.D.), the formulations of the synthesized peptides were confirmed using matrix‐assisted laser desorption/ionization‐time‐of‐flight mass analysis (MALDI‐TOF MS) on a microflex (Bruker Daltonics K.K., Billerica, MA, USA).

Peptide synthesizer MultiPep CF & MicroColumn (Intavis, Köln, Germany) was used to synthesize IGF with DOPA‐containing sequence (IGF‐DOPA). The low‐density resin for EGF‐DOPA synthesis was also used. The amount of the reagents was adjusted carefully to 0.5 and 1.0 mmol for normal and double coupling, respectively. The reaction times of 20 and 45 min for standard and extended times, respectively, for normal coupling and 45 and 60 min for standard and extended times, respectively, for double coupling were decided based on the machine monitoring of the deprotection process (Figure S2b, Supporting Information). After purification using HPLC equipped with a YMC‐Triart C18 column (250 × 4.6‐mm I.D.), the formulations of the synthesized peptides were confirmed using MALDI‐TOF MS on a microflex (Bruker Daltonics K.K.).

Subsequently, IGF‐DOPA was refolded. IGF‐DOPA was dissolved in the solubilizing buffer [50‐mm MES (pH 6.0), 1‐m NaH_2_PO_4_, 6‐m guanidine hydrochloride, and 10‐mm 2‐mercaptoethanol]. The IGF‐DOPA solution was then diluted using the refolding buffer [50‐mm MES (pH 6.0), 200‐mm NaCl, 1‐mm EDTA, 0.2‐mm oxidized glutathione, and 1‐mm reduced glutathione]. After refolding, the IGF‐DOPA solution was replaced with a buffer with 50‐mm MES (pH 6.0) and 10% (v/v) glycerol. Then the sample was concentrated via centrifugation and freeze‐dried for biological activity assays.

The formulations of the synthesized growth factor derivatives were confirmed using MALDI‐TOF MS (Figure S2E,F, Supporting Information).

### Binding Assay

The binding affinity of the synthesized peptides and growth factors to material surfaces was measured at 25 °C using QCM‐D (Meiwafosis Co., Tokyo, Japan). First, the buffer was allowed to run until the baseline stabilized. Then, the sample was pumped into the measuring system at 65 µL min^−1^ for 15 min. Subsequently, the substrate was washed using the running buffer and then Milli‐Q water. At least three measurements were performed and the average value was calculated.

After binding EGF‐DOPA to the Ti plates, the EGF moiety on the EGF‐DOPA layer was detected. The surfaces were blocked with 0.5% normal goat serum (Abcam, Cambridge, MA, USA) and incubated with a primary antibody against EGF (Rabbit anti‐hEGF, R&D systems, Minneapolis, MN, USA) for 1 h at room temperature. After three washes with PBS, the plates were incubated with an Alexa Fluor 488 anti‐rabbit antibody (Invitrogen, Carlsbad, CA, USA) and examined under an AxioObserver fluorescence microscope equipped with an AxioCam MRc5 camera (Carl Zeiss Co., Ltd, Göttingen, Germany).

The thickness of the growth factors bound on the surfaces was measured using an M‐2000UI ellipsometer (J.A. Woollam Co., Lincoln, NE, USA) in the spectral range of 245–1500 nm at three incident angles (50°, 60°, and 70°).

The morphology of the sample surface was observed using an MPD‐3D atomic force microscope (Asylum Research, Inc., Goleta, CA, USA). The images were captured in the AC (noncontact) mode using an atomic force microscope tip (NCH‐W, NanoWorld AG, Neuchatel, Switzerland) in dry atmospheric pressure conditions.

### Cell Culture and Assays

The cell lines of NRK49F, NIH3T3, MC3T3‐E1, and A431 were procured from the Japanese Collection of Research Bioresources Cell Bank. NRK49F, NIH3T3, and A431 cells were cultured in Dulbecco's modified Eagle's medium (DMEM, FUJIFILM Wako Pure Chemical Industries, Ltd., Osaka, Japan) supplemented with 5% fetal bovine serum (FBS, MP Biomedicals, LLC., Illkirch, France) and 1% penicillin–streptomycin (Nacalai Tesque, Inc., Kyoto, Japan) at 37 °C with 5% CO_2_. MC3T3‐E1 cells were cultured in the minimum essential medium Eagle‐alpha modification (MEM*α*, FUJIFILM Wako Pure Chemical Industries) supplemented with 1% penicillin–streptomycin (Nacalai Tesque, Inc., Kyoto, Japan) and 10% FBS at 37 °C with 5% CO_2_. The cells were then washed using 5 mL of PBS (pH 7.4, Nacalai Tesque, Inc.), harvested using 0.25% trypsin containing 1‐mm ethylenediaminetetraacetic acid for 3 min at 37 °C, and suspended in culture media for in vitro examination.

EGF‐DOPA and IGF‐DOPA's mitogenic activity was investigated. EGF‐DOPA or IGF‐DOPA was bound to the substrate surfaces and washed thrice with PBS. Then, the cells were seeded on the surfaces and cultured with 5% CO_2_ at 37 °C. Cell proliferation was analyzed with the WST‐8 assay using a Cell Counting Kit‐8 (CCK‐8, Dojindo, Kumamoto, Japan). In short, the CCK‐8 solution was added to the cell culture medium at the ratio of 1:10 to incubate at 37 °C in a humidified atmosphere with 5% CO_2_ for 3 h. Then, the absorbance of the medium was measured at 450 nm using a V‐550 spectrophotometer (JASCO, Tokyo, Japan). Statistical analysis was performed using analysis of variance.

Osteogenesis in MC3T3‐E1 cells was induced by adding 25‐µg·mL^−1^ ascorbic acid and 5‐mm glycerophosphate to the cultured medium. Cell differentiation was studied using the Alizarin Red S staining quantification assay. The cell culture medium was removed, and the MC3T3‐E1 cells were washed with PBS and fixed with 4% paraformaldehyde for 15 min at room temperature. After washing the cells with Milli‐Q water, 40‐mm Alizarin Red S (FUJIFILM Wako Pure Chemical Industries) was added to each well and incubated for 30 min at room temperature. After the dye was removed, the cells were washed with Milli‐Q water five times. The dye was extracted by adding 10% acetic acid to each well and incubating for 30 min at room temperature. The solutions were collected in 1.5‐mL tubes, heated at 85 °C for 10 min, incubated on ice for 5 min, and centrifuged at 20 000 g for 15 min. The supernatants were transferred to new tubes, mixed with 10% ammonium hydroxide, and measured at 405 nm.

The phosphorylation of EGFR triggered by growth factors was studied. The cells were seeded on the surfaces bound with EGF‐DOPA and incubated for 2, 4, and 12 h before immunofluorescence staining. The cells were fixed with 2% paraformaldehyde (Wako Pure Chemical Industries) for 15 min, permeabilized with 0.2% Triton X‐100 (FUJIFILM Calbiochem, La Jolla, CA, USA) for 5 min, blocked with 0.5% normal goat serum (Abcam, Cambridge, MA, USA), and incubated with primary antibody against phosphorylated EGFR (Santa Cruz Biotechnology, Santa Cruz, CA, USA) for 1 h at room temperature. After three washes with PBS, the cells were incubated with Alexa Fluor 488‐conjugated anti‐rabbit IgG antibody (Invitrogen). The surfaces were mounted using VectaShield mounting media containing DAPI (Vector Laboratories, Burlingame, CA, USA) to visualize nuclei and examined under an AxioObserver fluorescence microscope equipped with an AxioCam MRc5 camera (Carl Zeiss).

For confocal laser scanning microscopy, EGF‐DOPA was immobilized on glass coverslips. HeLa cells were starved with an overnight incubation in DMEM without serum before being seeded on the surfaces with EGF‐DOPA and cultured in serum‐free DMEM for 1 h. Then, the cells were fixed with 4% paraformaldehyde for 15 min, permeabilized with 0.2% Triton X‐100 for 5 min, and blocked with 5% solution of the ECL Prime blocking reagent (Cytiva, Marlborough, MA, USA). The phosphorylated EGFR of the cells was stained as mentioned above. The stained cells were mounted with ProLong Diamond Antifade Mountant with DAPI (Invitrogen). The distribution of the phosphorylated EGFR was observed using a TCS SP8 confocal laser scanning microscope (Leica Microsystems, Wetzlar, Germany), and vertical cross‐sectional views were obtained.

Phosphorylation assays of cell lysates were performed as follows. DMEM was removed at 0.5, 1, 2, and 12 h after incubation, and A431 cells were washed thrice with PBS and extracted with a lysis buffer [50‐mm Tris‐HCl (pH 7.0), 1‐mm EDTA, 150‐mm NaCl, 1% NP40, 10‐mm NaF, and 1‐mm Na_3_VO_4_]. Insoluble material was removed via centrifugation at 20 000 g for 15 min at 4 °C. Protein phosphorylation was analyzed via Western blotting using the anti‐phosphotyrosine (Millipore), anti‐EGFR (Santa Cruz Biotechnology), and anti‐ß‐actin (Cell Signaling Technology, Danvers, MA, USA) antibodies. The intensity of the bands was quantified with CS Analyzer software (Atto Corporation, Tokyo, Japan).

### Statistical Analysis

Data are shown as means ± standard deviation of three replicates unless specified. Graphs were plotted using Microsoft Excel.

## Conflict of Interest

The authors declare no conflict of interest.

## Supporting information

Supporting Information

## Data Availability

Research data are not shared.
