# Supplementary material for: Versatile Mitogenic and Differentiation‐Inducible Layer Formation by Underwater Adhesive Polypeptides
Source: Adv Sci (Weinh). 2021 Jun 26;8(16):2100961. doi: 10.1002/advs.202100961 (PMC8373149; doi:10.1002/advs.202100961)
Supplement: Supplementary file 1 — Supporting Information [file ADVS-8-2100961-s001.pdf]

WILEY-VCH

© 2021 Wiley-VCH GmbH

ADVANCED SCIENCE
Open Access

Supporting Information

for *Adv. Sci.,* DOI: 10.1002/advs.202100961

Versatile mitogenic and differentiation-inducible layer formation by underwater adhesive polypeptides

*Seiichi Tada, Xueli Ren, Hongli Mao, Yun Heo, Shin-Hye Park, Takashi Isoshima, Liping Zhu, Xiaoyue Zhou, Reiko Ito, Shino Kurata, Megumi Osaki, Eiry Kobatake, and Yoshihiro Ito**

Supporting Information

Versatile mitogenic and differentiation-inducible layer formation by underwater adhesive polypeptides

*Seiichi Tada, Xueli Ren, Hongli Mao, Yun Heo,Shin-Hye Park, Takashi Isoshima, Liping Zhu, Xiaoyue Zhou, Reiko Ito, Shino Kurata, Megumi Osaki, EiryKobatake, and Yoshihiro Ito**

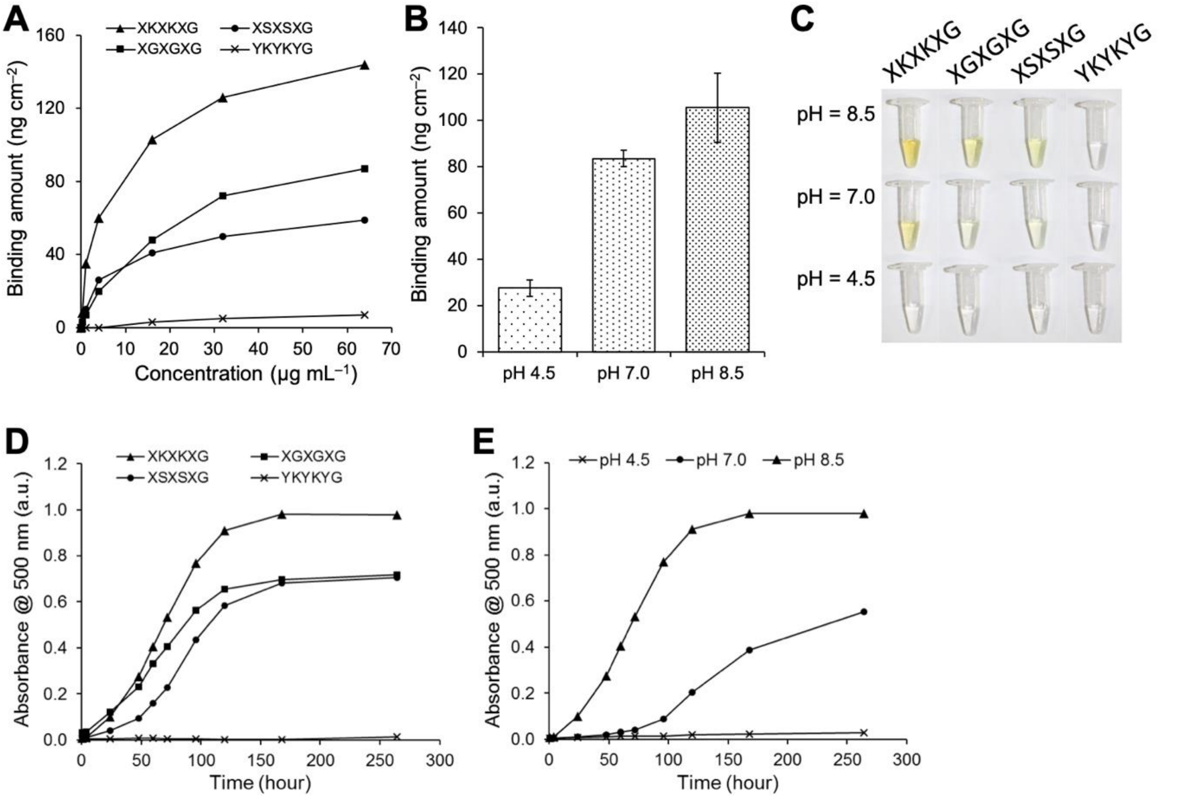

Figure S1. Binding affinity and turbidity analysis of DOPA peptides. A) Binding amount of the synthesized peptides on Ti surfaces at different concentrations measured by QCM-D. The pH value of the peptide solution was 8.5. The value is calculated from single QCM-D measurement. B) Binding amount of the synthesized XKXKXG peptide under different pH values measured by QCM-D. The concentration of the peptide solution was 16 μgmL^{-1}. Data were shown as means ± s. d. (n = 3). C) Physical appearances of the synthesized peptide solutions (2 mg mL^{-1}) after being incubated at room temperature for 7 d under different pH values. D, E) Time course of the turbidity of (D) different peptide solutions (2 mg mL^{-1}) and (E) XKXKXG solution (2 mg mL^{-1}) at different pH values. Data were plotted from single time course measurement.

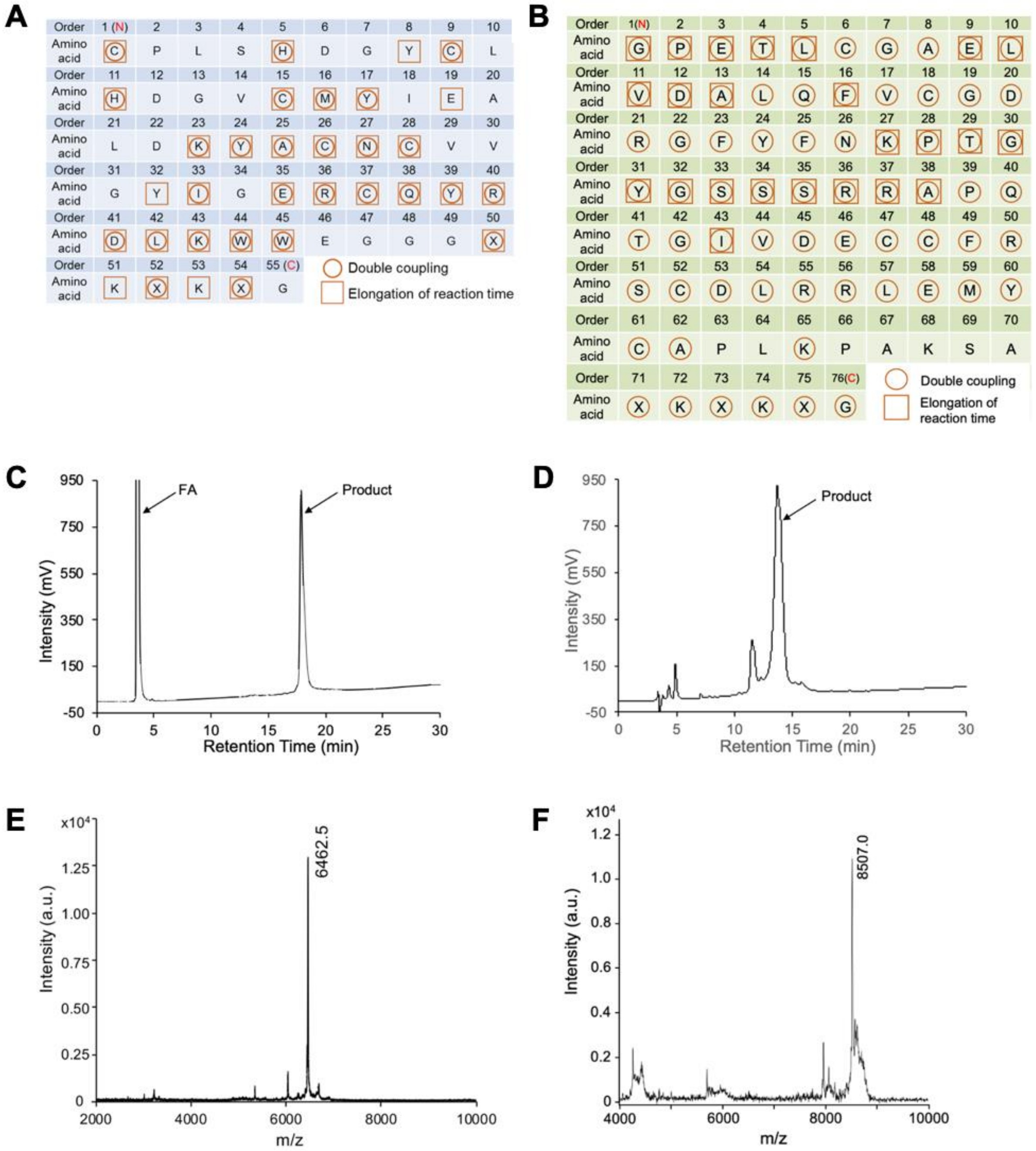

Figure S2. Synthesis of DOPA-conjugated growth factors. A,B) Reaction conditions for each amino acid conjugation of EGF-DOPA (A) and IGF-DOPA (B). C, D) HPLC analysis of EGF-DOPA (C) and IGF-DOPA (D). E, F) MALDI-TOF MS spectrum of the synthesized EGF-DOPA (E) and IGF-DOPA (F), respectively.EGF-DOPA: *m*/*z* $[M + H]^+$calcd for 6443.2 (Av.); found, 475.2593. IGF-DOPA: m/z $[M + H]^+$calcd for 8506.7 (Av.); found, 8507.0 (Av.)].

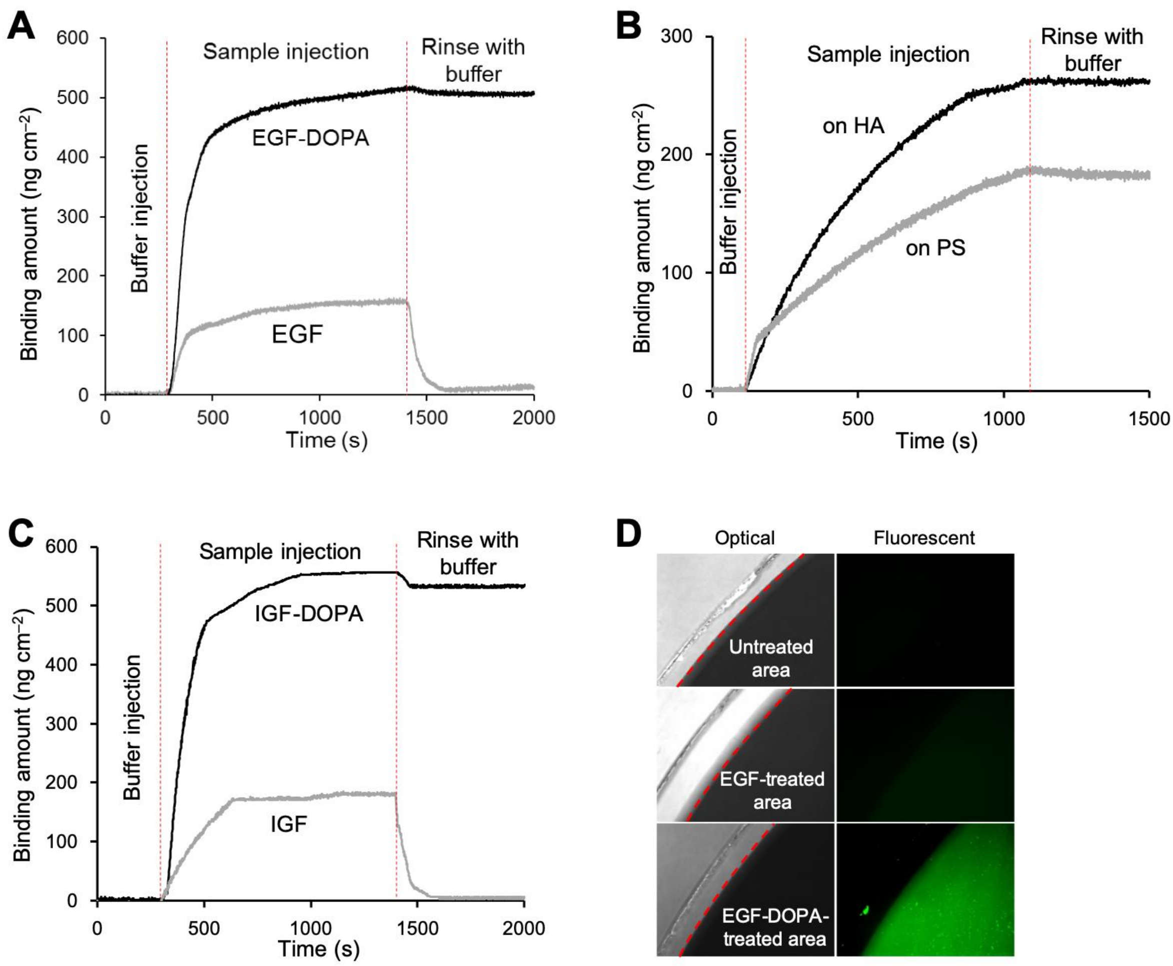

Figure S3. Binding affinity analysis of DOPA-growth factors. A, B) Binding curves of EGF-DOPA on Ti(A), on HA and PS surface (B) as measured using QCM-D.C) Binding curves of IGF-DOPA on the Ti surface measured using QCM-D. The pH value of the solution was 8.5. D) The immunofluorescence staining of Ti surfaces treated with EGF and EGF-DOPA using an anti-EGF antibody.

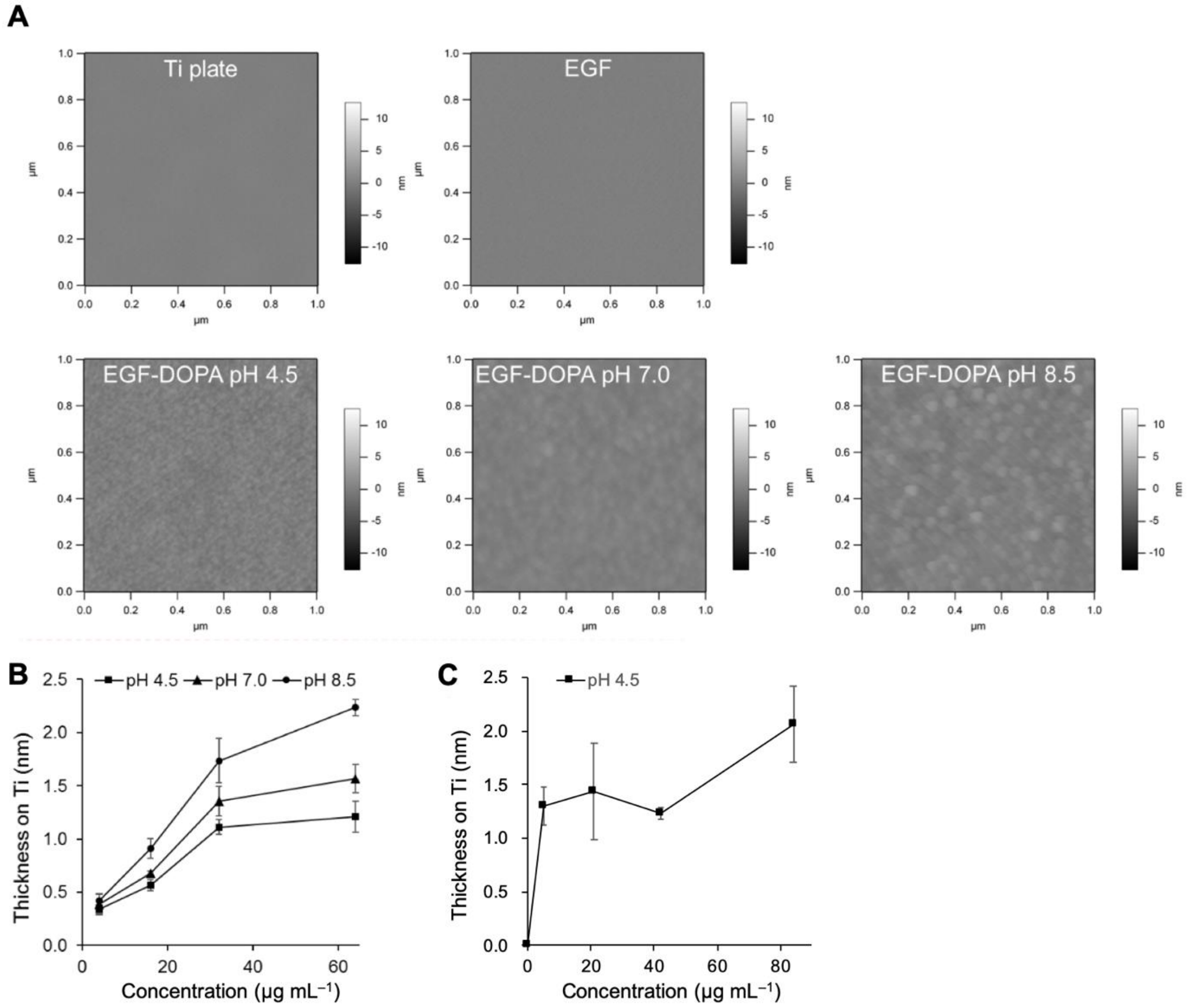

Figure S4. Surface analysis of DOPA-growth factor layers. A) AFM images of the bare Ti plate and Ti plates treated with EGF and EGF-DOPA under different pH values. The concentration of EGF and EGF-DOPA solution was 32 μg mL^{-1}. B, C) The thickness of EGF-DOPA (B) and IGF-DOPA (C) bound on Ti surfaces measured by Ellipsometry, respectively. Data were shown as means ± s. d. (n = 3).

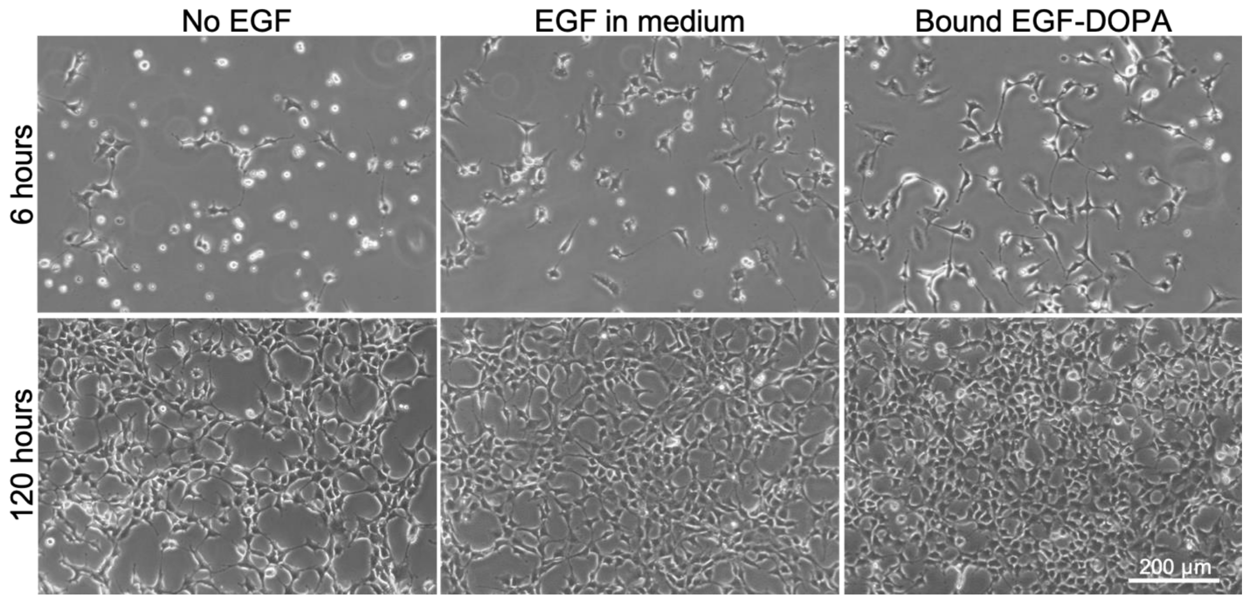

Figure S5. Phase-contrast images of cells cultured with soluble EGF in medium and bound EGF-DOPA on Ti surface (50 ng per well).
